# Buckling during drying of edible soft matter with cylindrical core–shell geometry

**DOI:** 10.1016/j.crfs.2025.101074

**Published:** 2025-05-27

**Authors:** R.G.M. van der Sman, Michele Curatolo, Luciano Teresi

**Affiliations:** a Wageningen Food & Biobased Research, The Netherlands; b Food Process Engineering, Wageningen University & Research, The Netherlands; c Roma Tre University, Italy

**Keywords:** Multiphysics, Large deformation mechanics, Drying, Simulation

## Abstract

This paper investigates the large deformation during the drying of cylindrical core–shell geometries, having a stiff skin and a soft core of gel-like material, representing food materials like fruits or vegetables. This particular geometry is inspired by our earlier study on the drying of broccoli stalks. The MRI imaging of broccoli drying had shown non-affine large deformations, which were explained by the presence of an elastic skin. We solve this multiphysics problem with COMSOL, where the mass transfer of water is strongly coupled to the momentum equation and the energy equation. The core–shell geometry necessitates a multi-domain formalism, which we have developed in another recent study.

Computer simulations show that the cylindrical core–shell system undergoes circumferential buckling if a critical buckling stress is imposed on the interface between core and shell. This critical stress depends on the ratio of shell thickness over total diameter τ/D, and the ratio of elastic moduli of core and shell $G_{h}/G_{s}$. The number of buckling modes is largely determined by the geometric ratio τ/D, but it is also slightly dependent on $G_{h}/G_{s}$. For relatively thick shells we also observe vertical buckling, next to circumferential buckling. The vertical buckling first shows a barrelling instability, which later transitions to snap-back buckling. These modes of vertical buckling bear large similarities with those observed via MRI imaging during broccoli drying.

As a final note, we have remarked that the presented model is a good starting point for modelling shape morphing of 4D printed foods, which is also governed by buckling instabilities as observed during our simulations.

## Highlights

• Large deformation mechanics is applied to drying of food.
• Buckling instability is simulated for cylindrical core–shell geometry.
• Scaling relation for the critical buckling stress is obtained.
• The number of buckling modes is largely dependent of ratio shell thickness/core diameter.

## Graphical abstract

**Figure:**
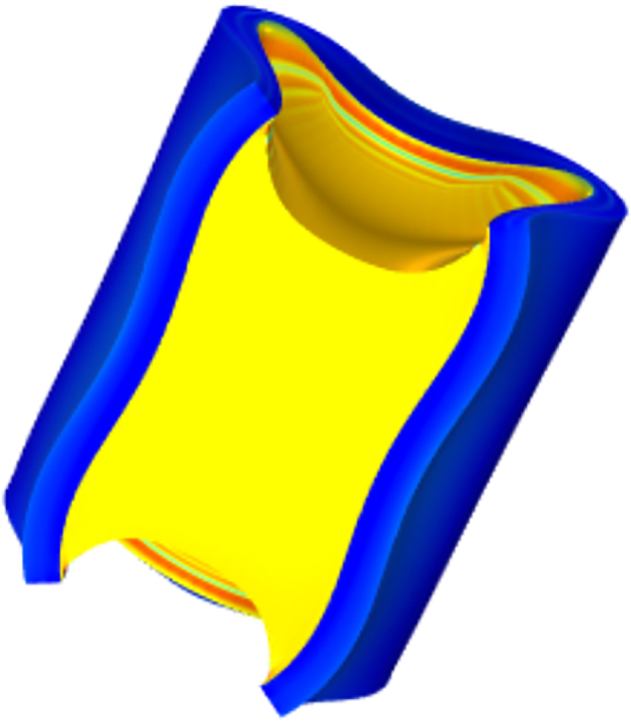

## 1 Introduction

Only in the last decade researchers are simulating the large mechanical deformation during drying of foods (Aregawi et al., 2014, Karunasena et al., 2014b, Liu et al., 2015, Singh et al., 2015, Gulati and Datta, 2015, Van der Sman, 2015b, Gulati et al., 2016, Rahman et al., 2018, Defraeye and Radu, 2018, Tuly et al., 2025). Most of these studies follow the Finite Element method as is the custom in solid mechanics. A few other studies follow particle-based methods (Karunasena et al., 2014b, Karunasena et al., 2014a), which modelled the deformation of a collection of cells at the microstructural scale of the tissue. However, with particle-based methods the modelling of moisture transport is quite involving. In this paper, we present a Finite Element method for describing large deformation of food materials during drying in a thermodynamic consistent way, for which we follow the framework developed by Suo and coworkers (Hong et al., 2008, Zhao et al., 2008, Hong et al., 2009, Marcombe et al., 2010). Their framework is an extension of the Flory–Rehner theory for inhomogeneous, anisotropic deformations. In our earlier papers we have used Flory–Rehner theory for describing water holding capacity of foods (Paudel et al., 2014, van der Sman et al., 2013, van der Sman, 2015, Cornet et al., 2021, Cornet et al., 2020) and the cooking of meat (van der Sman, 2012, van der Sman, 2013, Van der Sman, 2007). A limitation of the Flory–Rehner theory is the assumption of isotropic, uniform deformation. The framework by Suo and coworkers provides a consistent two-way coupling between the driving forces of mechanics and mass transport: the stress and chemical potential respectively, which are derived from a free energy functional. They are coupled via the pressure field, which is a result of the incompressibility of the food material. This framework has successfully been applied to describe the swelling of synthetic hydrogels (Hong et al., 2008, Zhao et al., 2008, Hong et al., 2009, Marcombe et al., 2010), and occasionally applied to dehydration of soft matter (Bertrand et al., 2016, Wang and Cai, 2015, Curatolo et al., 2018, Kang et al., 2021). In their application to synthetic hydrogels, the theory has been successful in describing phenomena in multilayer material, showing mechanical instabilities during swelling or dehydration, such as buckling and wrinkling (Weiss et al., 2013, Jin et al., 2015). Wrinkling is especially extensive if the material has a stiff outer surface and a soft inner part.

The application of the framework of Suo to deformation during food drying is scarce (Jin and van der Sman, 2022, van der Sman et al., 2024b, van der Sman et al., 2024a). These studies were limited to problems with spherical and cylindrical symmetries. In this study we present a full 3D model, applied to multi-layered food materials. Many fruits and other vegetables can also be regarded as multilayered soft materials, which exhibit intriguing deformations during drying (Yin et al., 2008, Liu et al., 2015). As such, we have shown that broccoli stalks are also multilayer materials, having a stiff elastic skin, and a softer inner tissue (Jin et al., 2012). Its deformation during drying has been observed with MRI. This earlier study has inspired us to perform the current study. From the MRI images, we have observed intriguing shape changes, which seemingly show first a barrelling instability, followed by a snap-back instability — which can be interpreted as vertical buckling. In our earlier study, we only examined the vertical cross-section, and thus no information on horizontal/circumferential buckling is available.

In general, core–shell geometries with perfect initial spherical or cylindrical shapes can exhibit regular buckling patterns if subject to dehydration. The regular buckling patterns resemble the wrinkling observed with real dried fruits and vegetables. In this study, we investigate in particular under which conditions buckling occurs for core–shell cylindrical geometries, similar to the broccoli stem. Knowledge of the critical conditions of buckling can give us more information about the asymmetry in mechanical properties between shell and core (Dai and Liu, 2014). Such knowledge can further be used for designing 4D printed multi-layered materials (Wu et al., 2019, Liu et al., 2019, Zhang et al., 2020).

4D printing is perceived as a novel way of producing attractive food products (Koirala et al., 2023). First, half-products are produced with simple planar geometries enabled by 3D-printing-inspired technologies. Subsequently, they undergo shape-morphing during post-processing like boiling (as in the case of pasta Tao et al., 2019, Tao et al., 2021, Di Palma et al., 2025) or oil-frying/air-frying/microwaving/drying/baking (as in case of crispy snacks Chen et al., 2023, Koirala et al., 2025). 4D food printing has the promise of reduction of amount packaging material, and the production of the simple half-product is easier to scale up than direct 3D printing of end-products with complex shapes (Koirala et al., 2023, Di Palma et al., 2025). However, there exist currently no numerical models via which one can predict what will be the resulting shape (Chen et al., 2022). The model presented in this paper can be a basis for the development of such predictive models.

**Fig. 1:**
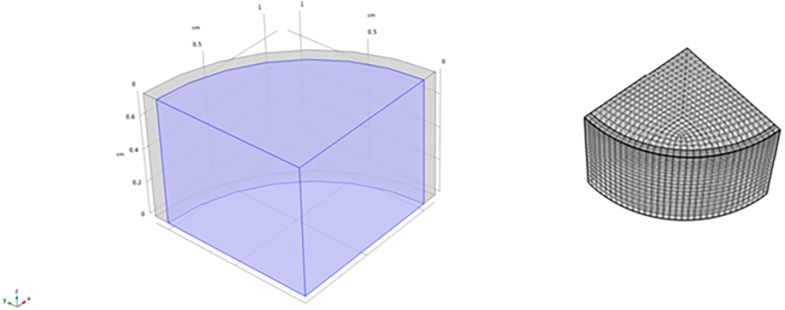
Left pane: Initial configuration of the core–shell geometry: a soft finite cylinder (purple), with an elastic skin (grey). Top and bottom surfaces are cutting planes, partly exposed to the inner soft tissue. The flat vertical planes are symmetry planes, imposing 4-fold rotation symmetry around the z-axis. Right pane: The mesh used for the Finite Element model.

## 2 Theory

### 2.1 State variables & kinematics

We assume that in the initial state, the food object has a perfect cylindrical shape of finite height. The outer curved surface is a thin elastic skin, with an elastic modulus larger than that of the soft inner tissue. The skin is still able to hold some water and allows for the diffusion of moisture to escape via the outer surface. The top and bottom surfaces of the cylinder are exposed to the soft inner tissue, as they constitute cutting surfaces. A sketch of the initial configuration is shown in Fig. 1.

In solid mechanics, it is custom to describe the large deformation with respect to a non-moving reference frame $B_{ref}$ (Hong et al., 2008). The position of a material point in this reference frame $B_{ref}$ is given by X, but due to deformation the material point has moved to a new position in the current frame ($B_{\tau }$): x=f(X,t)=X+u(X,t), with u the displacement vector. Via the deformation gradient tensor F=∇f=I+∇u (with I the identity tensor), one can compute how geometric elements transform from the reference frame $B_{ref}$ to the current frame $B_{\tau }$. We consider the transformation of a volume element $dv_{ref}$, a surface element $da_{ref}$, and a normal vector $m=n_{ref}$ from the reference frame $B_{ref}$ to those (dv,da,n) in the current frame $B_{\tau }$:

(1) $$dv=J\;dv_{ref}\;,\;da=\left|F^{*}\;m\right|\;da_{ref}\;,\;n=\frac{F^{*}\;m}{\left|F^{*}\;m\right|}$$

with the Jacobian J=detF, and the conjugate $F^{*}=J\;F^{-T}$. Below, we will use these relations for transformations of the governing equations from the current frame to the reference frame.

We like to note that in literature there is still a debate what is the correct reference state for polymer gels (Urayama et al., 1996, Quesada-Pérez et al., 2011, Sakumichi et al., 2022). Yet, it is stated that in principle the choice of a reference state is arbitrary (Gurtin et al., 2010, Curatolo et al., 2024). Hence, often for synthetic polymers the dry state (without solvent) is taken as the reference state (Hong et al., 2009). However, in polymer physics, it is argued that the polymer fraction at preparation (with solvent present) must be taken as the reference configuration. Yet, food gels have often physical crosslinks, and consequently, there is no clearly defined preparation state.

Because we have a bilayer material (core–shell material), which is stress-free and uniformly swollen in the initial state, it can be shown that a dry configuration with zero stress and uniform deformation is physically not realizable. Hence, for our problem, it is more convenient to use the initial, free swollen configuration $B_{0}$ as the reference frame. The system attains its free swollen configuration in equilibrium with pure water, with water activity $a_{w}=1$. In the free-swollen configuration, the system has uniform deformation and zero stress.

Yet, (food) hydrogels the biopolymers in the gel network are elastically stretched in the initial free swollen configuration state (van der Sman, 2015). Consequently, in polymer physics one takes as the reference frame the condition, where the biopolymers are unstretched. In the initial state, the biopolymers have a uniform, isotropic stretch $\lambda_{i}=\lambda_{0}$. In our earlier paper (Van der Sman, 2015a) we have shown that for many food materials, there is a universal value $J_{0}=\lambda_{0}^{3}=3/2$, independent of the degree of crosslinking. There we have defined the polymer volume fraction in the free-swollen configuration as $\phi_{0}$, and the polymer volume fraction in the unstretched configuration as $\phi_{ref}=\lambda_{0}^{3}\phi_{0}$.

As our system is a bilayer core/shell system with each layer having a different crosslink density, we think it is convenient to define the deformation with respect to taking the initial free-swollen configuration $B_{0}$ but define the elastic deformation with respect to the unstretched configuration $B_{u}$. Hence, the elastic deformation $F_{e}$ is defined following a multiplicative decomposition of F:

(2) $$F=F_{e}F_{u}$$

Thus in the initial state F=I, and $F_{e}=\lambda_{0}I$, and thus it follows that $F_{u}=\lambda_{0}^{-1}I$.

**Fig. 2:**
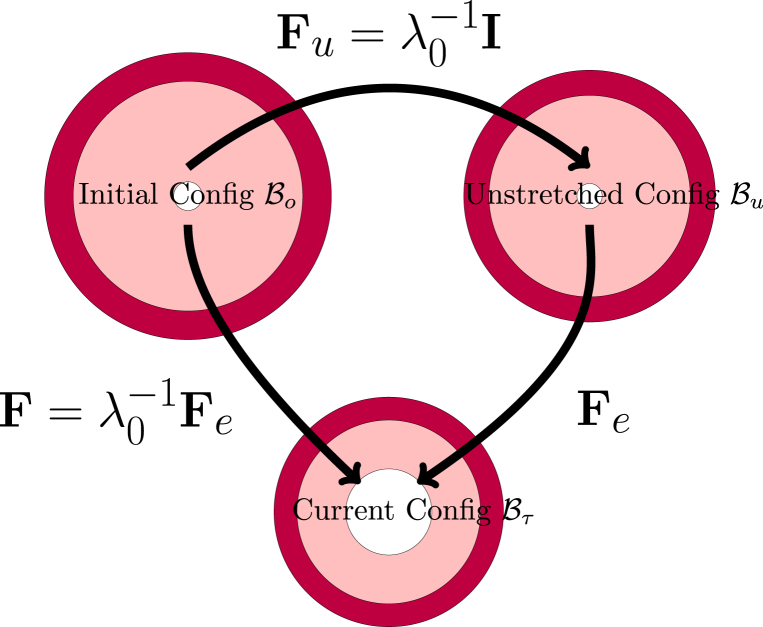
Definition of the various configurations for defining the deformation. The initial configuration is used as the domain frame, defining the deformation F to the current (deformed) configuration. The elastic deformation $F_{e}$ is defined via the reference configuration. Note, that in all 3 configurations, the skin and core have matching deformations at their interface.

A similar multiplicative decomposition of F is commonplace in the field of plastic deformation (Gurtin et al., 2010), where the total deformation has both inelastic (plastic) and elastic contributions, with the latter defined with respect to an intermediate stress-free configuration (Simo, 1988). In our problem, the unstretched configuration frame serves as our intermediate configuration, from which we compute the elastic deformation. Note, that core and shell have different unstretched reference configurations, as they differ in $\phi_{ref}$. This multiplicative decomposition and its connection to the three defined coordinate frames is nicely illustrated in Fig. 2. Thus, we have the following transformations:

• F maps the deformation of material points from the initial configuration $B_{0}$ to the current configuration $B_{\tau }$, with $\det F=J=\phi_{0}/\phi$,
• $F_{u}$ maps from the initial configuration $B_{0}$ to the intermediate unstretched configuration $B_{u}$, with $\det F_{u}=J_{u}=\phi_{0}/\phi_{ref}=1/J_{0}$, and
• the elastic deformation $F_{e}$ maps from the intermediate unstretched configuration $B_{u}$ to the current configuration $B_{\tau }$, with $\det F_{e}=J_{e}=1/\overset{\sim }{\phi }=\phi_{ref}/\phi$,

Thus, instead of the immediate mapping F, one can do this also in two steps: via $F_{u}$, and $F_{e}$. The stress will be a function of the elastic deformation, which will be computed as: $F_{e}=FF_{u}^{-1}=FF_{0}=\lambda_{0}F$. Note, $\overset{\sim }{\phi }=1/J_{e}$ we have used earlier in our papers about Flory–Rehner theory (Van der Sman, 2015a).

### 2.2 Balance laws

We formulate the problem using Cartesian coordinates, which allows for the development of wrinkles, and other non-affine deformations. Assuming cylindrical symmetry prohibits their development, as these mechanical instabilities are symmetry-breaking. Following the common approach in solid mechanics for hydrogels (Hong et al., 2008), we solve the problem via the Finite Element method using the weak formulation. Before giving the weak form, we state the balance equations in the strong form in both the co-moving Lagrangian frame, and the reference frame.

#### 2.2.1 Mass balance of water

As a state variable for the mass balance of water, we use the molar concentration c, which is in line with implementations of models by Suo (Hong et al., 2009, Hong et al., 2008). It is convenient to relate c to the volume fraction of water, $\phi_{w}$, as we will use the Flory–Huggins theory. Their relation is:

(3) $$\phi_{w}=c\;\Omega$$

with Ω the molar volume of water. The amount of water in the initial (fully swollen) configuration is $c_{0}$ or $\phi_{w,0}=1-\phi_{0}$.

Let $D_{t}=\partial_{t}+\nabla \cdot v$ be the material derivative, with v the spatial velocity defined by the time derivative of the deformation field $\overset{˙}{u}$. For the co-moving Lagrangian frame, the mass balance for the water in the core and skin is (Hong et al., 2008):

(4) $$D_{t}\;c=-\nabla \cdot j_{w}=\nabla \cdot \frac{D_{s}\;c}{R_{gas}\;T}\;\nabla \mu_{w}$$

where $j_{w}$ is the diffusive water flux given by the generalized Darcy’s law, $D_{s}$ is the self-diffusivity of water, and $\mu_{w}$ is the chemical potential (in units of [J/mol]).

The chemical potential can be decomposed into two contributions:

(5) $$\mu_{w}=\mu_{w,mix}+p\Omega$$

p is the hydrostatic pressure in the solvent, which is a consequence of the incompressibility of the hydrogel material. $\mu_{w,mix}$ is the so-called mixing contribution, and it is derived from Flory–Huggins theory, as we have shown earlier for the case of several vegetables like broccoli (van der Sman et al., 2013, Jin et al., 2014). For simplicity we assume an constant Flory–Huggins interaction parameter, with χ=0.5. Thus, the chemical potential equals:

(6) $$\mu_{w}/RT=\log\left(\phi_{w}\right)+\phi_{s}+\chi \phi_{s}^{2}$$

In the Finite Element solver the problem is solved in the initial reference frame. As a state variable we use the concentration per unit of volume in the reference frame: $c_{d}=cJ$, with J=det(F) the shrinkage factor. The strong form in the reference frame is:

(7) $$\partial_{t}c_{d}=-\nabla_{X}\cdot h_{w}$$

with the molar flux defined as:

(8) $$h_{w}=JF^{-1}j_{w}$$

Using $j_{w}=-M\nabla \mu_{w}=-MF^{-T}\nabla_{X}\mu_{w}$, and $M=D_{s}c/RT$, it follows:

(9) $$\partial_{t}c_{d}=\nabla_{X}\overset{\sim }{M}\nabla_{X}\mu_{w}$$

with the contravariant mobility equal to:

(10) $$\overset{\sim }{M}=JF^{-1}MF^{-T}$$

#### 2.2.2 Balance of forces

We assume that inertia forces are negligible and external forces are null; the balance of forces in the current configuration writes as:

(11) ∇⋅σ=0

where σ is the actual stress (Cauchy stress).

The strong formulation in the reference frame uses the first Piola stress S, which relates to σ via:

(12) $$S=\sigma F^{*}$$

The strong form in the reference frame is simply:

(13) $$\nabla_{X}\cdot S=0$$

Independent of the choice of the reference configuration the elastic energy is expressed in terms of tensor invariants of Cauchy $C_{e}=F_{e}^{T}F_{e}$. We use the NeoHookean model:

(14) $$\psi_{e}=\frac{1}{2}G\left(I_{1}-3\right)$$

with $I_{1}=tr\left(C_{e}\right)$. The related true stress equals:

(15) $$\sigma_{e}=\frac{G}{J_{e}}F_{e}F_{e}^{T}-pI$$

The elastic stress in the material frame equals (mapped from intermediate to current frame):

(16) $$S_{e}=\sigma_{e}F_{e}^{*}$$

The mechanical part of the elastic stress is $S_{e,m}=\partial \psi_{e}/\partial F_{e}=GF_{e}$. Mind that $S_{e}$ also contains a pressure term.

We require the reference stress (mapped from reference to current frame):

(17) $$S=S_{e}F_{u}^{*}=\sigma F^{*}=S_{e,m}F_{u}^{*}-pF^{*}$$

It is multiplied with $F_{u}^{*}$ as stress acts on the surfaces of the volume elements. Surface area between configurations are mapped via the adjugate $F_{i}^{*}$. As $F_{u}^{*}=\lambda_{0}^{-2}I$, the reference stress tensor becomes.

(18) $$S=G\lambda_{0}^{-2}F_{e}-pF^{*}=G\lambda_{0}^{-1}F-pF^{*}$$

#### 2.2.3 Energy balance

For the drying problem, the model needs to be complemented with an energy balance, which reads:

(19) $$D_{t}e=-\nabla \cdot q$$

$e=\rho_{eff}c_{p,eff}T$ is the thermal energy density, with T the temperature. $T_{ref}$ is an arbitrary reference temperature for defining the enthalpy. Often, it is convenient to take $T_{ref}$ = 273 K. $\rho_{eff}$ is the effective density of the vegetable, and $c_{p,eff}$ is the effective specific heat. In the model we will take their product as a single parameter, denoting the volumetric heat capacity, $C_{eff}=\rho_{eff}c_{p,eff}$, which has the same value in either current or reference configuration.$C_{eff}$ depends on composition via:

(20) $$C_{eff}=\phi_{w}\rho_{w}c_{p,w}+\phi_{s}\rho_{s}c_{p,s}=\phi_{w}C_{w}+\phi_{s}C_{s}$$

The heat flux q has both convective as diffusive contributions (van der Sman, 2013): $q=q_{diff}+q_{conv}$. The diffusive contribution is due to thermal conduction, and follows Fourier’s law:

(21) $$q_{diff}=-k\nabla T$$

k is the thermal conductivity. The convective contribution is linear in the diffusive mass flux, $j_{w}$ carrying enthalpy away from the (co-moving) control volume:

(22) $$q_{conv}=C_{w}\Omega j_{w}$$

(23) $$q=C_{w}\Omega j_{w}-k\nabla T$$

The convective contribution is often neglected in studies concerning thermosensitive hydrogels (Drozdov, 2014, Ding et al., 2016, Brighenti and Cosma, 2022), but is correctly described in Li (2009).

The energy balance in the reference frame reads (Li, 2009):

(24) $$\partial_{t}JC_{eff}T=-\nabla_{X}\cdot Q$$

Note, the prefactor J accounts for the change of $C_{eff}$ from the current frame to the reference frame. The heat flux reads (Moya et al., 2021):

(25) $$Q=-\overset{\sim }{K}\nabla_{X}T+C_{w}J_{w}T$$

$\overset{\sim }{K}$ is the contravariant tensor of the thermal conductivity:

(26) $$\overset{\sim }{K}=F^{-1}kF^{*}$$

### 2.3 Boundary conditions

The mass flux at the boundary is given by:

(27) $$j_{w,ext}\cdot n=-M\nabla \mu_{w}\cdot n=\beta \left(c_{v,surf}-c_{v,ext}\right)$$

with n the normal vector (in the current frame), $c_{v}$ the vapour concentration in the air, β the mass transfer coefficient. $c_{v,surf}$ is the molar vapour concentration in the boundary layer, which is in equilibrium with the liquid phase at the boundary:

(28) $$c_{v,surf}=a_{w}c_{sat}\left(T\right)$$

$a_{w}$ is the water activity, related to the chemical potential: $\mu_{w}=RT\log\left(a_{w}\right)$. $c_{sat}$ is the saturated vapour concentration, related to the saturated vapour pressure:

(29) $$c_{sat}\left(T\right)=\frac{p_{sat}\left(T\right)}{RT}$$

The saturated vapour pressure is temperature dependent, following well-known relations like the Clausius–Clapeyron relation.

$c_{v,ext}$ is often specified via the relative humidity $RH_{air}$:

(30) $$c_{v,ext}=RH_{air}c_{sat}\left(T_{air}\right)$$

The heat and mass transfer at the boundary are coupled to each other due to the evaporative cooling effect, which is expressed as:

(31) $$q_{ext}=h\left(T-T_{ext}\right)+j_{w,ext}\nu_{w}\left[\Delta H_{evap}+c_{p,v}T\right]$$

$\Delta H_{evap}\left(T\right)$ is the (molar) latent heat of evaporation.

The relation between heat and mass transfer coefficient is the Lewis relation:

(32) $$\beta =\frac{h}{\rho_{air}c_{p,air}}$$

$c_{p,air}$ is the specific heat of air (J/kg.K), and $\rho_{air}$ is the mass density of air.

We now consider the transformation of the boundary conditions to the material frame, which follows from the precondition that the fluxes in either frame are equal if integrated over their surface area:

(33) $$J_{w}\cdot mdA=j_{w}\cdot nda$$

m is the normal vector in the material frame. Via the Nanson formula, we obtain:

(34) $$n=\frac{F^{*}m}{\left|F^{*}m\right|}$$

To explain the Nanson formula we define the adjugate of the normal vector m in the reference frame:

(35) $$\overset{ˆ}{n}=m^{*}=F^{*}m$$

The adjugate needs to be normalized, to get a normal vector:

(36) $$n=\frac{m^{*}}{\left|m^{*}\right|}$$

Using $J_{w}=F^{*}j_{w}$ we obtain:

(37) $$dA=\frac{da}{\left|F^{*}m\right|}$$

Consequently:

(38) $$J_{w,ext}=j_{w,ext}\left|F^{*}m\right|$$

A similar transformation holds for the heat flux:

(39) $$Q_{ext}=q_{ext}\left|F^{*}m\right|$$

### 2.4 Weak formulations

For the momentum balance, we use the gradient of the displacement field as a test function. The weak form on the reference configuration is:

(40) $$\int_{B}S\cdot \nabla \;\overset{\sim }{u}\;dV=0$$

The incompressibility of the tissue will be enforced via a weak constraint in the energy functional:

(41) $$\int_{B}\overset{\sim }{p}\left(J-\frac{\phi_{0}}{\phi }\right)dV$$

The test function for the incompressibility constraint is the pressure field: $\overset{\sim }{p}$.

The weak form of the mass balance is obtained using $c_{d}$ as the test function:

(42) $$\int_{B}\left({\overset{\sim }{c}}_{d}\partial_{t}c_{d}-J_{w}\nabla_{X}{\overset{\sim }{c}}_{d}\right)dV=\int_{\partial B}{\overset{\sim }{c}}_{d}J_{w,ext}dA$$

Note, that the right-hand side represents the Robin-type boundary condition at the outer boundary.

Using the temperature T as a test function, the weak form of the energy balance becomes:

(43) $$\int_{B}\left(JC_{eff}\overset{\sim }{T}\partial_{t}T-Q\nabla_{X}\overset{\sim }{T}\right)dV=\int_{\partial B}\overset{\sim }{T}Q_{ext}dA$$

We will implement the model in Finite Element, which assumes that the state variable is continuous across domain interfaces. For the mass, momentum, and energy balances, the chosen state variables (test functions) are the displacement u, the molar concentration $c_{d}$, the pressure p, and temperature T. However, the molar concentration $c_{d}$ will not be continuous, but the continuity condition holds for the chemical potential. Via a weak constraint, we will impose the continuity of chemical potential:

(44) $$\int_{\partial B}{\overset{\sim }{c}}_{d}D_{\mu }\left(\mu_{h}-\mu_{s}\right)dA$$

$\mu_{h}$ and $\mu_{s}$ are the chemical potential of the hard shell, and the soft core, respectively. ${\overset{\sim }{c}}_{d}$ is the test function of the mass balance. $D_{\mu }$ is a kinetic parameter determining the rate of equalizing the chemical potential difference $\mu_{h}-\mu_{s}$.

### 2.5 Implementation

The model is implemented in the Finite Element package COMSOL, using the weak formulation for all balance equations. The mesh we have used is shown in Fig. 1, showing that the meshing is rather uniform. The skin is resolved with 2 elements in the radial direction, and the core is resolved with 20 elements in the radial direction. In the circumferential direction the skin is resolved with 32 elements. In the vertical direction we used 20 elements. For all balance equations we use quadratic Lagrangian element, only the incompressibility condition linear (Discontinuous Lagrangian) elements are used. For numerical stability it is required that the order of the elements for the pressure field p are an order lower than for the deformation field (u) (the so-called Ladyzhenskaya–Babuska–Brezzi condition). We used the PARDISO for direct solving of the matrix equation, and the implicit BDF scheme for time integration using adjusted absolute and relative tolerances. The balance equations are solved in a segregated fashion.

### 2.6 Buckling theory

Classical buckling theory was developed for thin shells by Landau and Lifshitz (Landau et al., 2012). The theory is later formalized as incremental theory, which has been applied to cylindrical core–shell geometries (Zhao and Zhao, 2017). Due to the mathematical complexity of the incremental theory, we restrict ourselves to insights obtained from shell theory.

The dimensionless number governing buckling is (Moulton and Goriely, 2011):

(45) $$Bu=\frac{G{\left(\tau /R\right)}^{n}}{\Delta p}$$

with Δp the compressive pressure over the spherical shell. τ is the thickness of the shell, which is much smaller than the radius R. G is the elastic modulus. Buckling occurs at a critical value $Bu_{cr}$. The scaling exponent depends on the geometry. For spherical shells holds n=2, while for a long cylinder under plain strain n=3 (Papadakis, 2008). The scaling exponent appears to be sensitive to thickness variations (Gusic et al., 2000), leading to n=2.5. For cylinders with moderate lengths, the buckling is governed by Zou and Foster, 1995, Lo Frano and Forasassi, 2008:

(46) $$Bu=\frac{G}{\Delta p}{\left(\tau /R\right)}^{5/2}\left(R/L\right)$$

The critical buckling pressure should be measured at the inner wall (Curatolo et al., 2023), or rather at the core–shell interface in our case (Yin et al., 2008).

For thin shells, the number of buckling modes depends on the ratio τ/R (Gusic et al., 2000). For core–shell geometries τ/R largely determines the number of buckling modes, but with a slight dependency on the ratio of elastic moduli $G_{h}/G_{s}$ (Yin et al., 2008, Cao et al., 2012, Liu et al., 2015). Also, the critical buckling pressure depends on both the ratio $G_{h}/G_{s}$ and τ/R (Yin et al., 2008, Liu et al., 2013). For spheroidal core/shell geometries, the aspect ratio H/R will also influence the buckling behaviour (Yin et al., 2008). It is expected that for H/R>3 the behaviour is analogous to cylindrical core–shell geometries (Yin et al., 2008). For a long cylinder a simplified ring model was derived, stating that the critical buckling pressure scales linear with τ/R, i.e. n=1 (Yin et al., 2009). For cylindrical geometries, one can observe circumferential buckling, potentially combined with axial buckling (Liu et al., 2019, Zhan et al., 2022). For the extreme of H/R≪1, the geometry is a thin circular plate, where buckling also occurs out of plane (Liu et al., 2013, Mehta et al., 2022). Similar buckling can also occur for annular bilayer plates, where a soft material is deposited on top of a more stiff material (Mehta et al., 2022).

Thus, for our short cylindrical core/shell geometry the height/radius ratio would also influence the buckling, but we restrict this study to a fixed ratio.

The literature studies on core–shell geometries usually use (homogeneous) growth (Yin et al., 2008) or swelling (Liu et al., 2019) to induce the buckling instability, but dehydration can lead to similar buckling behaviour (Yin et al., 2008, Liu et al., 2013). However, shrinkage is often implemented similar to growth, but in the reverse direction using a homogeneous growth factor smaller than unity (Yin et al., 2008, Liu et al., 2015). For growth/swelling/dehydration-induced buckling, little scaling relations for critical buckling stress and the number of buckling modes are reported.

In the model below we describe moisture transport realistically as occurs during drying when processing vegetables or fruits, leading to moisture gradients during shrinkage. Consequently, buckling is expected to start at the rims of the shell, which propagates towards the centre line at z=0, similar to the buckling during swelling of a pumpkin-like geometry, where buckling starts at the two poles of the pumpkin geometry (Wu et al., 2019). The number of buckling modes will remain constant during the propagation of the sinusoidal perturbations to the centre line (equator) of the geometry.

**Fig. 3:**
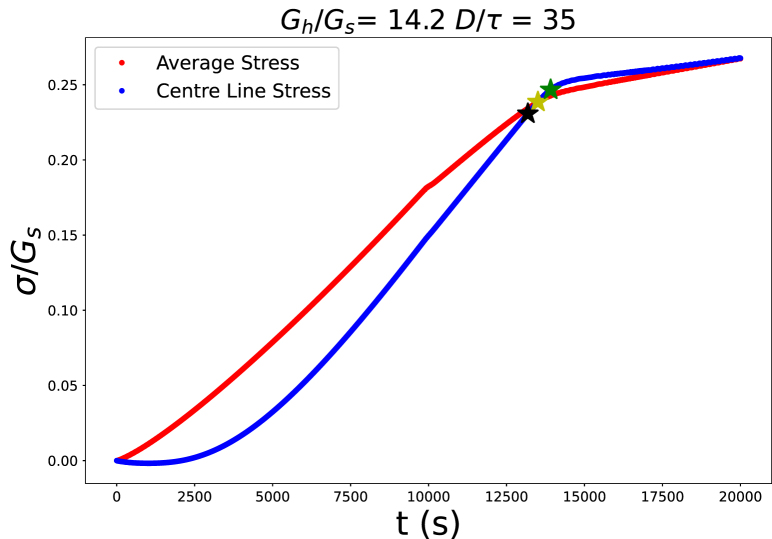
Evolution of average stress on the core–shell interface, and at its centre line at z=0. Simulations are performed for $G_{h}/G_{s}=14.2$ and τ/D=1/35. (For interpretation of the references to colour in this figure legend, the reader is referred to the web version of this article.)

## 3 Results

Simulations are performed using COMSOL, with all equations programmed via the weak form. Simulations are stopped when a quasi-steady value in the stress between core and shell domains is observed in the post-buckling stage. In our drying simulations, we have varied the ratios of the elastic moduli, $G_{h}/G_{s}$, and thickness to diameter, τ/D, but kept the ratio of height and radius H/D constant, due to the long duration of simulations.

The material of the core and shell are assumed to follow the Flory–Huggins theory for their moisture sorption, similar to the case of broccoli. The Flory–Huggins interaction coefficient is set to $\chi_{1}=0.8$. The outer diameter of the cylinder is D, the height of the cylinder is H, and the elastic modulus of the soft core is fixed at the value $G_{s}$. Environmental conditions were set at air temperature $T_{ext}=46\;{}^{\circ}C$, and relative humidity RH=48%. The initial product temperature is $T_{init}=20\;{}^{\circ}C$. For drying outside air is heated, such that the absolute humidity in the air is not changed. Hence, the molar vapour concentration in the environment is $c_{v,ext}=RH_{air}c_{v,sat}\left(T_{init}\right)$, with $c_{v,sat}$ the saturated vapour concentration at a given temperature.

We assumed a heat transfer coefficient $h_{ext}=10$ W/m${}^{2}$.K, and the mass transfer coefficient is linked via the Lewis relation: $\beta_{ext}=h_{ext}/\rho_{air}c_{p,air}$. The outer skin of the shell is assumed to have a waxy layer, which is captured in an elevated mass transfer coefficient $\beta_{skin}=\beta_{ext}/4$. Simulations were performed up to times $18000<t_{e}<35000$ s.

An animation of a typical simulation is shown in the Supplementary Material, with colours indicating the value of the Jacobian J. One can observe that significant moisture loss occurs at the cut surfaces at the top and bottom. The stiff skin resists deformation, making the cut surface concave. Eventually, buckling is initiated at the top of the stiff shell, which propagates downwards. After some time, the whole shell is buckled. For the major part of the shell the displacement is independent of the axial position. Only, at the cut surfaces, the shell bends more inwardly.

Following the buckling theory of thin shells we expect that buckling occurs if the pressure at the core–shell interface exceeds a critical value. We have monitored the average stress at this interface, and its centre line at z=0 (the horizontal symmetry plane). We show the typical evolution of these stresses in Fig. 3. The stresses are compressive, meaning $\sigma_{rr}<0$. However, we plot only the magnitude of the stresses. We observe that there is initially a more-or-less linear growth of the average stress, which is due to a mismatch in the deformation of the core and shell. As indicated in the animation, the soft core shrinks more than the stiff shell. The stress levels off due to the event of buckling — which is a mechanism of stress relief. The cylinder will continue to dry without further stress build at the core–shell interface.

**Fig. 4:**
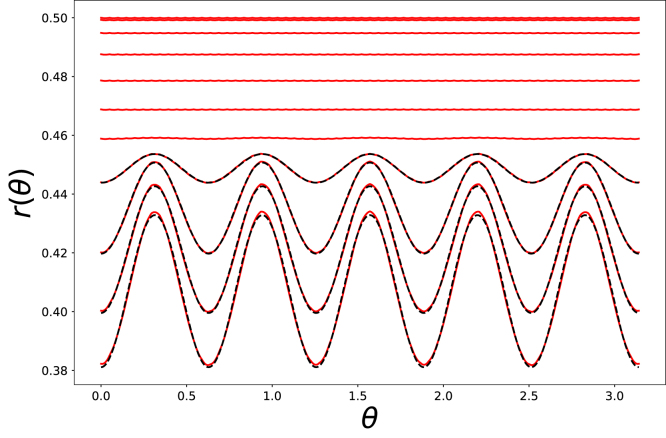
Radial coordinates of the centre line (at z=0) of the core–shell interface as function of the polar angle θ, at equidistant times during simulations of cylinder drying with $G_{h}/G_{s}=14.2$ and τ/D=1/35.

We have also followed the radial deformation at the core–shell interface. An example of its evolution is shown in Fig. 4. We observe that the onset of buckling is aroundt=14000 s, which is the moment that the average stress at the centre-line levels off, as shown in Fig. 3. The deformation shows that the mode of buckling is m=2: $r\left(\theta \right)=\overset{¯}{r}+\delta \cos\left(m\theta \right)$. With further drying both the average radius $\overset{¯}{r}$ decreases, while the amplitude of the perturbation δ increases.

**Fig. 5:**
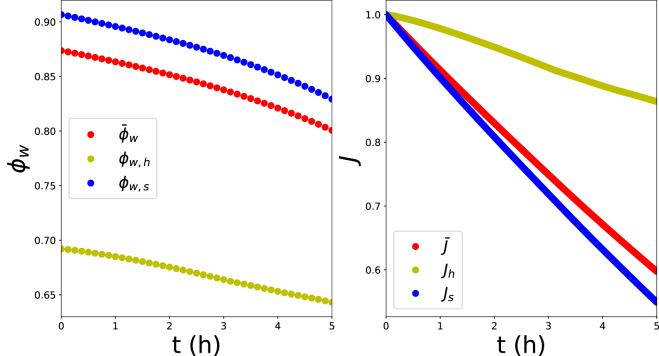
Left pane: Evolution of water volume fraction $\phi_{w}$ of (a) averaged over the whole domain ${\overset{¯}{\phi }}_{w}$, (b) averaged over the hard shell domain $\phi_{w,h}$, and (c) averaged over the soft core domain $\phi_{w,s}$. Right pane: Evolution of shrinkage factor J, average over whole domain ($\overset{¯}{J}$), hard domain ($J_{h}$), and soft domain ($J_{s}$). Simulations are performed for $G_{h}/G_{s}=14.2$ and τ/D=1/35.

The trends in volume fraction of water $\phi_{w}$ and the shrinkage factor J are shown in Fig. 5 for the case of τ/D=1/25 and $G_{h}/G_{s}=14$. We show the values averaged over the whole cylindrical domain, and the hard shell and soft core. We observe that the hard shell shrinks much less than the soft core. We like to note, that there is no exceptional/critical events happening in the drying behaviour at the moment of buckling (t≈ 4 h). Criticality happens in the strain mismatch between core and shell, which is a consequence of the drying tough. The transition happens only in the mechanical domain of the system, and there is no consequent redistribution of moisture, as evident by the smooth curves of the shrinkage factors. The overall shrinkage is largely determined by the shrinkage of the core. On the other hand, we observe a comparable decrease in the volume fraction of water, albeit the two domains have different initial values due to their differences in crosslink density. Hence, both domains are subject to moisture loss due to the outside drying conditions.

**Fig. 6:**
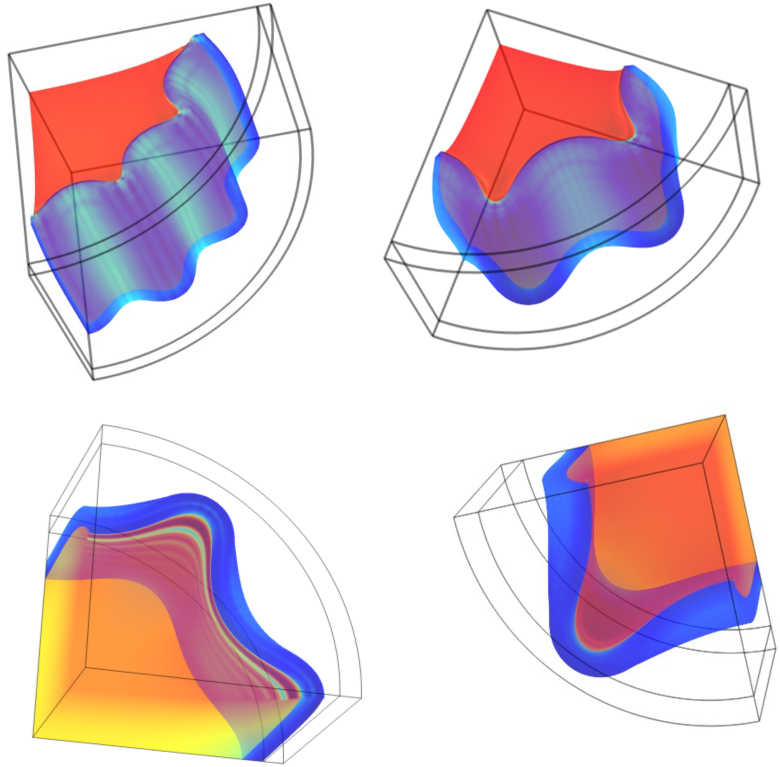
Examples of different buckling modes for a drying core–shell cylinder. Simulations are performed for (a) $G_{h}/G_{s}=9.0$ and τ/D=1/40, (b) $G_{h}/G_{s}=11.0$ and τ/D=1/30, (c) $G_{h}/G_{s}=14.0$ and τ/D=1/25, and (d) $G_{h}/G_{s}=20.0$ and τ/D=1/20, showing m=6, m=4, m=3, and m=2 buckling modes respectively.

Depending on the ratio τ/D one can observe different buckling modes, as shown in Fig. 6, displaying the overview of buckled shapes at the end of the simulation for a range of $G_{h}/G_{s}$ and τ/D. Note, that due to our choice of simulating only a quarter of the domain (for faster computation) the lowest mode of buckling is m=2, as observed for τ/D=1/20. For thinner shells, we observe higher modes of buckling, with m=6 for τ/D=1/40. Surprisingly, we observe m=3 for τ/D=1/25. Hence, the symmetry boundary conditions at the xz and yz planes allow for odd values of m.

We have performed a quantitative analysis of the critical buckling stress as a function of $G_{h}/G_{s}$ and τ/D, using the fitting procedure as detailed in the Supplementary Material. We have fitted the perturbations of the radial coordinate of the outer surface: $r\left(\theta \right)=\overset{¯}{r}+\delta \cos\left(m\theta \right)$ at equidistant time intervals. After buckling δ>0. We have determined the moment of buckling $t_{0}$, where δ/R=5e−4. The local stress at the interface between core and shell at z=0 at $t=t_{cr}$ is taken as the critical buckling stress. We have determined the outer radius ${\overset{¯}{r}}_{cr}$ and shell thickness $\tau_{cr}$ at $t=t_{cr}$.

For many of our simulations, this critical stress is about equal to the stress at the intersection point between the average stress and the local stress. We take this stress as another characteristic of stress. Another characteristic stress is the maximum of the local stress. Before buckling there is a monotonic increase of the local stress, which eventually shows a lower stress growth rate. The time with the minimum in the second derivative (i.e. the absolute value of the second derivative shows a peak) demarcates this transition. The three characteristic stresses are indicated in Fig. 1 of the Supplementary Material. For all three characteristic stresses, we have determined the corresponding average radius of the outer surface $\overset{¯}{r}$, and the thickness of the shell τ.

We have examined if these characteristic stresses follow a scaling law with $\tau_{cr}/\overset{¯}{r}$, and with τ/D, the initial ratio of thickness and diameter of the core–shell system. The various scalings of characteristic stresses against thickness over diameter ratio are shown in the Supplementary Material. The best scaling results are obtained with the characteristic stress at the transition between stress growth rates, as indicated with the green star in Fig. 3. This critical stress is indicated with $\sigma_{max}$.

**Fig. 7:**
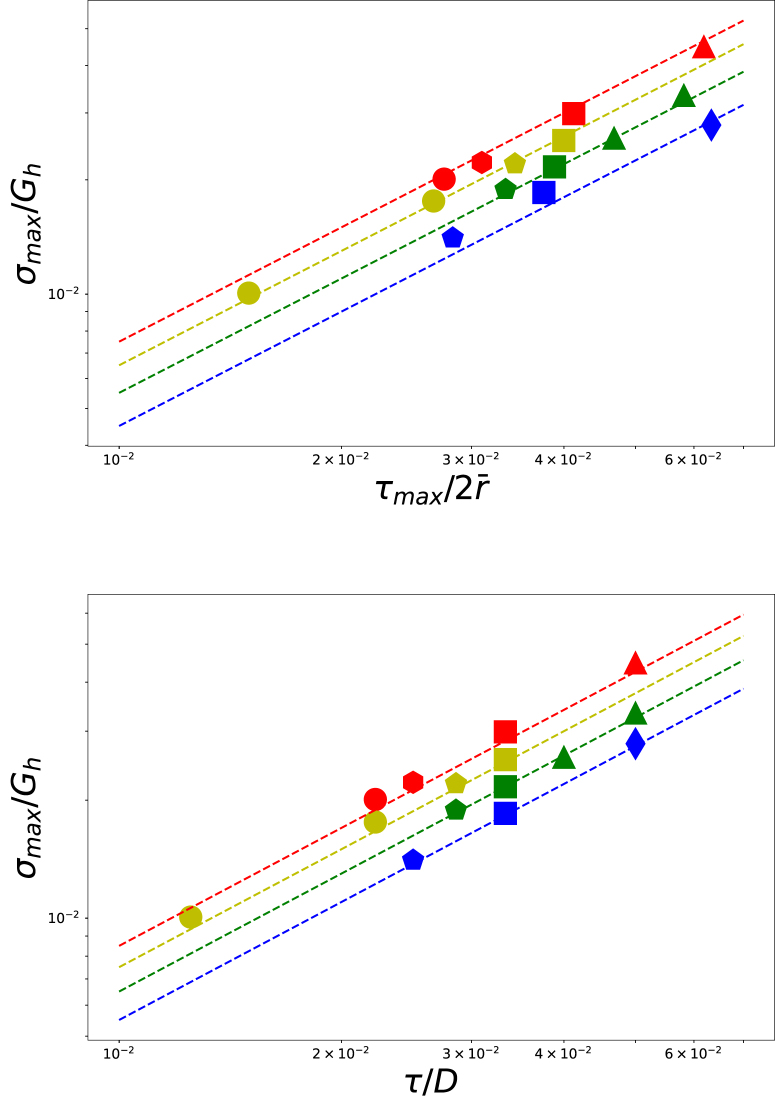
Scaling of the critical buckling pressure at the core–shell interface as a function of the ratio of thickness and diameter, at the moment of buckling (top pane) and at the initial stage, with t=0 (bottom pane). Dashed lines show the power law with n=1. Different colours indicate different values of $G_{h}/G_{s}$ (rygb for {9,11,14,20}). Symbol shape indicate the buckling mode (diamond m=2, triangle m=3, square m=4, pentagon m=5, hexagon m=6, and circle m>6.

We have plotted the critical buckling stress $\sigma_{max}/G_{h}$ versus τ/D in Fig. 7. Also, we show scaling laws with n=1 as dashed lines, with the intercept depending on $G_{h}/G_{s}$. Buckling modes are largely determined by τ/D, with a minor dependency on $G_{h}/G_{s}$.

**Fig. 8:**
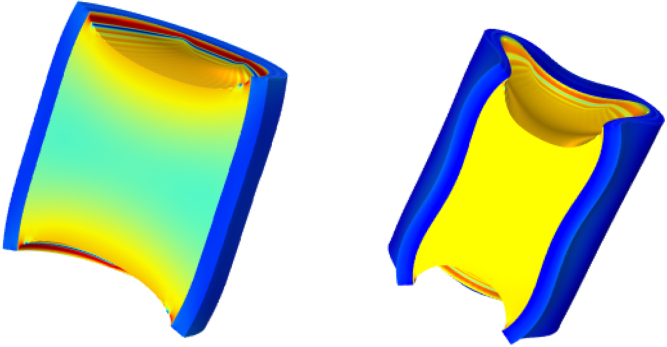
Onset of vertical buckling occurring during simulation of drying with $G_{h}/G_{s}=20$, and τ/D=1/15. The first image (left) shows a barrelling instability at t≈12000s, followed by a snap-back instability, shown in the second image (right) at t=29000s. Next, to the vertical buckling, circumferential buckling with m=2 has occurred. Colour indicates the value of J. At the core–shell interface at the top and bottom cut surfaces one can observe some local oscillations, which are probably numerical artefacts that do not affect the overall behaviour.

The onset of vertical buckling is observed for the extreme case of τ/D=1/15 and $G_{h}/G_{s}=20$, as shown in Fig. 8. For this case, the simulation is performed on half of the geometry to allow also for lower order buckling (m=1). We observe that first, a barrelling instability happens at t≈12000s, where the middle slightly bulges out. Subsequently, at continued drying, we observe a snap-back vertical buckling, where the middle gradually snaps inwardly. Simultaneously, circumferential buckling with m=2 is happening. We have also included an animation of this combined vertical and circumferential buckling. The barrelling instability and subsequent snap-back buckling have been observed also for finite hyperelastic cylindrical shells under axial compression (Chen and Jin, 2020). This behaviour of barrelling instability and snap-back buckling is also evident from our previous MRI imaging of drying broccoli stalks (Jin et al., 2012).

Hence, the current model is an important step towards realistic modelling of deformation during drying of vegetables. However, the broccoli stalk show little circumferential buckling, while in the model the circumferential buckling is dominant over vertical buckling. We infer that circumferential buckling is likely suppressed by fibres present in the skin, which stiffen the broccoli skin in the circumferential direction. To be more realistic we probably need to extend the model with strain hardening by the fibres, as we have performed earlier (Van der Sman, 2015b). Furthermore, the current implementation becomes instable if simulations are continued far beyond the buckling. We wonder if the stress levels under these conditions are realistic. We note that the current model assumes the vegetable purely elastic, while it has been shown that many vegetables and fruits are actually viscoelastic (Ozturk and Takhar, 2020, Ozturk and Takhar, 2021, Abedi and Takhar, 2022). Viscoelastic relaxation reduces the stress levels as compared to the pure elastic case, and probably the instability can be avoided.

## 4 Conclusions

In this paper, we have presented a multiphysics model, which is able to describe circumferential buckling occurring during the drying of a cylindrical core–shell geometry, representing food material like a broccoli stalk. The buckling happens if the stress in the plane interfacing the core and shell domains exceeds a critical value. This critical buckling stress is shown to be a function of the ratios τ/D the thickness over radius, and $G_{h}/G_{s}$, the ratio of elastic moduli of the stiff shell and soft core. The number of buckling modes largely depends on τ/D, but there is a slight dependency on $G_{h}/G_{s}$ too. For relatively thick skins we have observed vertical buckling too, happening simultaneously with circumferential buckling. The vertical buckling is first showing barrelling instability, which eventually leads to snap-back buckling.

This type of model can be used for the description of drying of many other fruits and vegetables, or other food materials if the mechanical properties are known as a function of moisture and temperature. Moreover, we think this type of model can be a first step towards the prediction of shape morphing of 4D printed food materials, as the shape morphing is often based on buckling. We have done some initial calculations with our model, where H/D≪1, which shows indeed well-programmable shape-morphing as induced by out-of-plane buckling.

## Declaration of competing interest

Two authors of the paper are guest editors of the special issue. Review is performed by independent co-editor.

## Data Availability

Data will be made available on request.

## Appendix A Supplementary data

MMC S1 Supplementary Material contains 2 videos showing the buckling during drying, and a graphical depiction of the fitting procedure.
