# Supplementary material for: Buckling during drying of edible soft matter with cylindrical core–shell geometry
Source: Curr Res Food Sci. 2025 May 27;10:101074. doi: 10.1016/j.crfs.2025.101074 (PMC12167106; doi:10.1016/j.crfs.2025.101074)
Supplement: MMC S1 — Supplementary Material contains 2 videos showing the buckling during drying, and a graphical depiction of the fitting procedure. [file mmc1.pdf]

Supplementary Material: Buckling during drying of edible soft matter with cylindrical core-shell geometry

R.G.M. van der Sman, Michele Curatolo, Luciano Teresi

May 5, 2025

Video material: animation of buckling

The file ShrinkingBroc.gif shows the animation of a typical circumferential buckling (m=4) during drying of a finite cylindrical core-shell geometry with $G_h/G_s = 11.0$ and $\tau/D = 1/30$. Simulation is performed on a quarter of the domain with symmetry boundary conditions at the x-z and y-z planes.

The file ShrinkingHalfBrocSnapBack.gif shows an animation of the simultaneous circumferential and vertical buckling of a finite cylindrical core-shell geometry with $G_h/G_s = 20$, and $\tau/D = 1/15$. Colors indicate the value of the Jacobian J. Simulation is performed with half of the actual domain, with symmetric boundary conditions at the x-z plane.

Depiction of fitting procedure

With figure 1 we show how we performed the fitting procedure to obtain characteristics of the buckling instability. We show the results for the simulation with $D/\tau = 35$, and $G_h/G_s = 14$. We start with fitting to the position of the outer surface the function $r(\theta, t) = \bar{r}(t) + \delta(t)\cos(m\theta)$ via the Fast-Fourier-Transform, as shown in the top pane. We fit a quadratic function to $\delta(t)$, and extrapolate to the moment that buckling occurs $\delta(t) \to 0$, as shown in the middle pane. The red dots indicate $\delta(t)$ and the red dotted line is the fitted quadratic function, and the zero point is indicated with the black star. From the time that $\delta(t) \to 0$ (t_{cr}) we estimate the $\bar{r}_{cr} = \bar{(}t_{cr})$, and

the thickness of the shell as that time $\tau_{cr} = \tau(t_{cr})$. In the bottom pane, we show how the average stress and the local stress at the interface between core and shell at $z = 0$ evolve with time. With symbols, we indicate the critical stress via the above fitting function (black star), the intersection point of the average and local stresses (yellow star), and the maximum local stress (green star). We have determined the times that these characteristic stresses occur and determined the outer radius and shell thickness at these times.

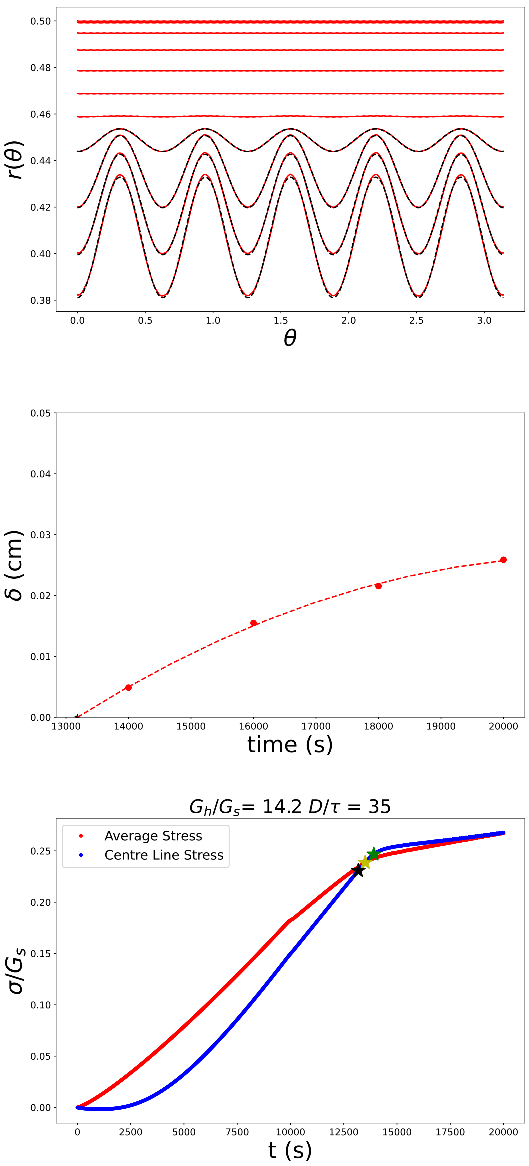

Figure 1: Intermediate results from the fitting procedure to obtain the critical stress(es). First, the perturbation δ) on $r(\theta)$ at various time intervals was fitted with Fast-Fourier-Transform (top pane). Secondly, as shown in the middle pane the perturbation amplitude δ is fitted as a function of time, and the zero-point is estimated (black star). The critical stress σ_{cr} is taken as the point where $\delta \to 0$, as shown by the black star in the bottom pane. Other characteristic stresses are the intersection between average stress and local stress on the centre line (yellow star), and the maximum stress at the centre line (green star).
